# Texture features explain the susceptibility of grapevine cultivars to *Drosophila suzukii* (Diptera: Drosophilidae) infestation in ripening and drying grapes

**DOI:** 10.1038/s41598-020-66567-9

**Published:** 2020-06-24

**Authors:** Lorenzo Tonina, Folco Giomi, Manuel Sancassani, Matteo Ajelli, Nicola Mori, Lara Giongo

**Affiliations:** 1 Università di Padova - Legnaro (Pd), Italy-Department of Agronomy, Food, Natural resources, Animals and Environment (DAFNAE), Legnaro, PD Italy; 2 Fondazione Edmund Mach - San Michele all’Adige (Tn), Italy - Genomics and Biology Fruit Crops Department, Research and Innovation Centre, San Michele all’Adige, TN Italy; 3 Università di Verona - Italy - Department of Biotechnology, Verona, Italy

**Keywords:** Invasive species, Plant physiology, Agroecology

## Abstract

Grapevine is a well-known host plant of the invasive pest *Drosophila suzukii*, but its susceptibility to pest oviposition and development greatly depends on the cultivar. To address environmental sustainability during viticultural zoning planning, new vineyard plantation and Integrated Pest Management programmes, it is essential to take pest pressure and cultivar susceptibility into account. To determine the different grapevine cultivars susceptibility to *D. suzukii*, we tested twelve widely spread cultivars during the ripening period. We also tested three cultivars during the drying period for raisin wine production. The infestation and emergence rates were consequently related to chemical and texture features of the berries to explain the role of skin and pulp characteristics in determining the nature of the susceptibility. Our results showed that susceptibility to *D. suzukii* infestation varies across cultivars. On ripening grapes, infestation is primarily influenced by skin and pulp firmness, elasticity and consistency. Suitability for egg development resulted mainly related to skin and pulp deformation. In a drying loft, infestation may also occur in relation to skin and pulp consistency. Lastly, we discuss the practical implication of the underestimated role of berry texture in *D. suzukii* oviposition and emergence success, in both ripening and drying grapes.

## Introduction

Newly introduced and invasive pests represent important challenges for crop production, and the magnitude of their detrimental effects should be accurately evaluated when a cultivar has to be selected. For grapevine, several differences in pest and disease incidence are reported according to cultivars and ripening stages^1–4^. Cultivar susceptibility to pests and diseases should thus be implemented within viticultural zoning programmes and in targeted Integrated Pest Management (IPM) strategies^5–7^. Differences among cultivars become relevant in viticulture because of a wide range of intrinsic physico-chemical characteristics, different ripening stages, grape uses, and wine destinations^8,9^.

Among grape pests, *Drosophila suzukii* Matsumura (Diptera: Drosophilidae) is a widespread and harmful species for ripe berries, due to its ability to lay eggs in healthy fruit close to the harvest period, the large number of generations per year, high reproductive capacity and wide host range^10,11^. Grapevine represents a host fruit for *D. suzukii*^12–16^, although it is affected by low oviposition, slow developmental rates, and limited survival to the adult stage (maximum 9%)^13,17–19^. Together with the direct damage caused by the larvae trophic activity, *D. suzukii* can facilitate *D. melanogaster* Meigen infestation^20^ and vehicle a large number of microorganisms, including acetic acid bacteria, favouring sour rot outbreaks^18,20–22^.

Infestations during ripening have been described in cultivars with soft skin^16,23,24^ confirming *D. suzukii* preference for fruits with thin skin and low penetrating resistance^18,25–31^. However, the skin resistance is only one aspect of the entire berry which is also characterized by a complex of anatomical and physical properties (e.g. skin firmness, pulp and berry consistency and elasticity). These properties, measured as mechanical response to applied forces, are used to characterize the cultivar differences of grapes and their quality^32–36^. The present study aims to test which among the anatomical and physical properties of the berry determines the higher cultivar susceptibility to infestation during the ripening period for all the winemaking productions. Understanding which cultivar and ripening stage are the most susceptible to oviposition would improve IPM practices and drive specific insecticide treatments and their optimal timing^13^.

In detail, this study was intended to identify the susceptibility to the infestation and development of *D. suzukii* in twelve highly valuable grape cultivars during the last phases of the ripening process and covering the entire harvesting period, by measuring the oviposition ability and emergence rate. In particular, the study aims to integrate a vast array of physical and mechanical proprieties related to berry texture and chemical features to identify the main drivers of susceptibility. For this reason, a penetrometer texture analysis was chosen to mimic the pest oviposition activity and its development.

In addition, grapes devoted to drying process, as for the production of valuable straw wines, may form a further substrate for *D. suzukii* oviposition and development, with a consequent detriment to the oenological process^37^. This drying process, which can last up to 100–120 days, is characterized by major changes of berry physical and chemical parameters. An accurate evaluation of the texture changes during the drying phases and their relationship with the incidence of pest infestation is needed to assess the vulnerability of these particular renewed traditional procedures. Three cultivars, suitable for drying process and devoted to the production of renowned raisin wines, were also analysed during postharvest, in order to test if the physio-chemical modifications that occur during the drying process would affect the success of *D. suzukii* infestation and development.

The proper characterization of cultivar susceptibility and analyses of environmental conditions are pre-requisite to design a rational *D. suzukii* control strategy within a wide grape growing area.

## Results

### Ripening grapes

#### Grape susceptibility to D. suzukii infestation

To evaluate the level of infestation we compared the percentage of infested ripening berries with the number of eggs laid per berry. Since these two indicators resulted highly correlated (n = 32, df=382, p-value < 0.0001, r^2^ = 0.50), the percentage of infested berries was used in all analyses as proxy for infestation level.

**Figure 1:**
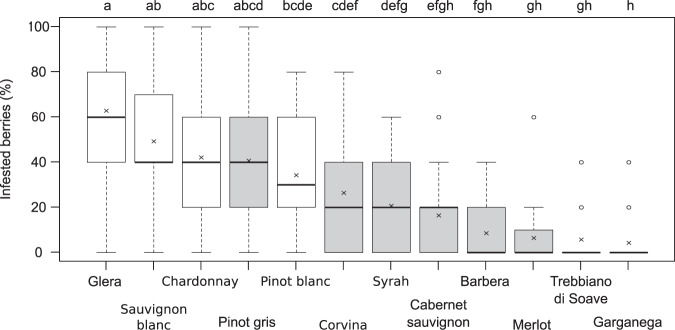
Level of infestation (percentage of infested berries) in the twelve tested cultivars over the whole period. The box edges enclose the first and third quartiles of the observations, the ends of the whiskers represent the 5th/95th percentiles, the solid line and cross indicate the median and mean respectively, the dots are the outliers. The different box colours indicate white-yellow (white) and blue-black (grey) cultivars.

Significant differences in infestation were found among the tested grape cultivars (ANOVA, Linear Model: F_11:307_ 21.95, p < 0.001) but not with collection time (F_1:5_ 0.36, p = 0.55) or the interaction between cultivar x time (F_11:307_ 1.22, p = 0.27; Fig. 1). The progress of maturation was also tested using chemical proxies to line up and monitor the different cultivars decoupling the observation from the collection date. However the analyses on sugar content, pH and total acidity showed no significant effects on infestation (F_1:82_ 0.03, p = 0.86 for the sugar content, F_1:82_ 2.47, p = 0.12 for pH and F_1:82_ 0.05, p = 0.11 for total acidity, see also supplementary Fig. S1) also considering the interaction between these parameters and cultivar (F_11:60_ 0.62, p = 0.81; F_11:60_ 0.62, p = 0.80; F_11:60_ 0.76, p = 0.67 for sugar content, pH and total acidity respectively), and further supporting the lack of a clear effect of time on infestation rate.

Emergence rate showed low values in all the tested cultivars (Table 1) reaching maximum values (over 10%) in Cabernet sauvignon, Garganega, Glera, and Sauvignon blanc. Five cultivars presented fewer than 4%. In Barbera and Trebbiano di Soave no adults were obtained.

**Table 1:** Total number of laid eggs, total number of emerged adults, and the resulting rate of emergence over the seven-week period.

Grape cultivars	Total number of laid eggs	Total number of emerged adults	Emergence rate (%)
Cabernet sauvignon (B)	82	10	12.2
Garganega (W)	19	2	10.5
Glera (W)	336	35	10.4
Sauvignon blanc (W)	245	25	10.2
Chardonnay (W)	255	13	5.1
Merlot (B)	31	1	3.2
Pinot blanc (W)	147	3	2.0
Syrah (B)	54	1	1.9
Corvina (B)	76	1	1.3
Pinot gris (B)	160	2	1.2
Barbera (B)	19	0	0.0
Trebbiano di Soave (W)	19	0	0.0

Cultivars are sorted by descending rate of emergence and white-yellow (W) and blue-black cultivars (B) are indicated.

#### Texture and chemical features

The physical and mechanical features of the berries, related to the texture analyses, varied over time among the twelve cultivars. Instead, the chemical features showed a more constant cultivar-specific signature during the ripening period (supplementary dataset).

#### Relationships among entomological-mechanical-chemical features

**Figure 2:**
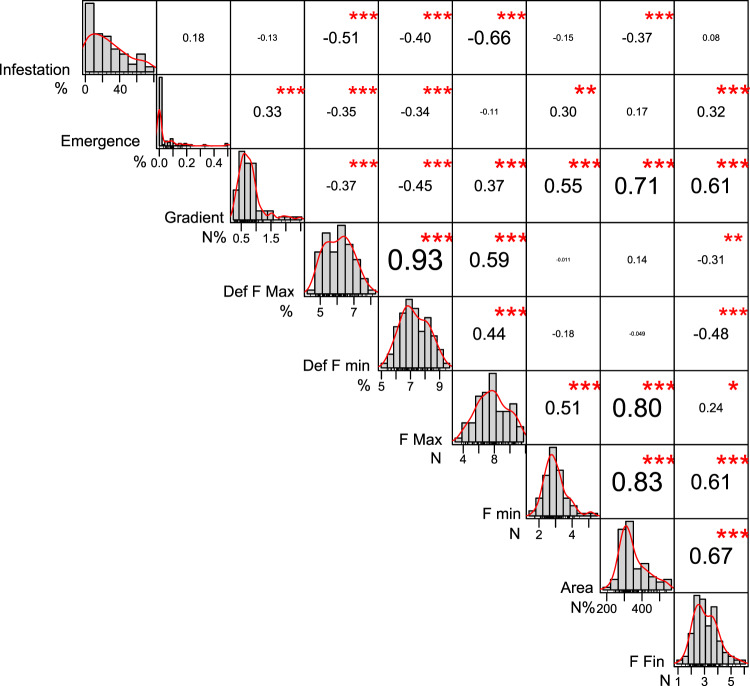
Correlation chart in the texture-entomological analysis for ripening grapes. The frequency distribution and kernel density estimation of each variable is shown on the diagonal. The value of the Pearson’s correlation (r) and the significance level (p-value) corrected for multiple comparisons as asterisks (***<0.001, **<0.01, *<0.05, <0.10) are shown at the top of the diagonal; the size of the numbers is an indicator of the correlation value. The data range of values and units of measurement are given at the bottom of the diagonal.

The correlation analyses revealed a sizeable influence of texture features of berries on the susceptibility of infestation and larval development (Fig. 2). In particular, infestation level declines when the maximum force increases (r = −0.66, P < 0.001; Fig. 2) and when deformations at both maximum and minimum force increase (r = −0.51 and r = −0.40 respectively, P < 0.001; Fig. 2). On this regard, the twelve cultivars accurately cluster along the curve of the correlation between maximum force and infestation level (supplementary Figure S2). Among the texture features, emergence rate is highly correlated with gradient, deformation at maximum force, deformation at minimum force, minimum force and final force (Fig. 2).

On the contrary, no significant effects of chemical parameters were discovered (supplementary Fig. S1).

**Figure 3:**
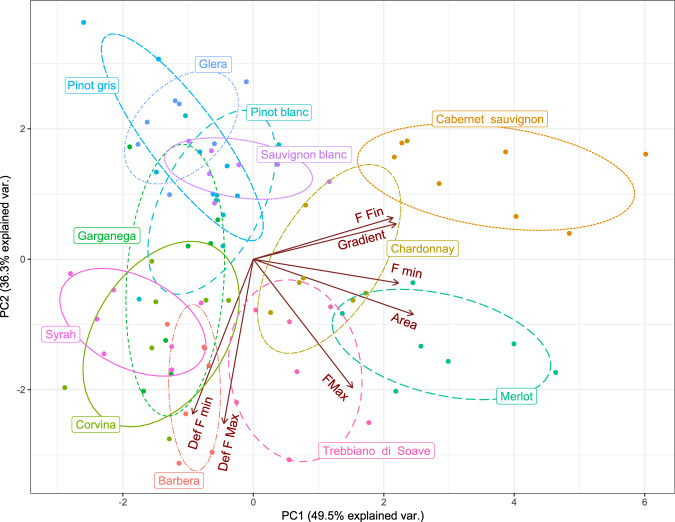
Spatial arrangement of mechanical texture features investigated in the PCA analysis for grapes in the ripening process. Ellipses group the individual values of the cultivars (95% confidence).

The grouping of the twelve cultivars is evident in PCA 2D-plot (Fig. 3), performed to depict the investigated mechanical texture variables, which explains with the first and second components 85.8% of total variability, thus very high. The spatial distribution of the twelve cultivars is consistent with the variables orientation imposed on the PCA plot. Sauvignon blanc, Glera, Pinot blanc, and Pinot gris are plotted in the area related to low values of internal forces in general, mainly maximum force. Merlot and Trebbiano di Soave are in the area characterized by high levels of maximum force.

Barbera, Corvina, Garganega and Syrah are characterized by high values of deformation, both at maximum and minimum force. Cabernet sauvignon is characterized by higher levels of final force and gradient, thus higher elastic properties, plotting in the low rate of infestation quadrant, defined by PC1.

Berry skin and pulp characteristics, derived from the specific mechanical texture analyses, are listed in Table 2 in order to summarize the level of infestation and emergence of the different cultivars in relation to the resulting phenotypic berry description.

**Table 2:** Summary of cultivar texture characteristics in relation to *D. suzukii* infestation and emergence level.

Grape cultivars	Level of infestation	Level of emergence	Skin*/pulp characteristics from texture profile	Berry characteristics
Barbera (B)	-	-	High deformation at maximum and minimum force	Highly elastic berries
Cabernet sauvignon (B)	+	++	High final force and high gradient	Firm pulp and low skin elasticity
Chardonnay (W)	++	+	Low maximum force, low deformation at maximum and minimum force	Soft pulp and skin
Corvina (B)	+	+	Variable mid-high maximum force, high deformation at maximum and minimum force	Variable skin firmness and consistent pulp
Garganega (W)	-	++	Mid-high maximum force, variable deformation at maximum and minimum force	Firm skin and variable pulp firmness
Glera (W)	+++	++	Low maximum force, low deformation at maximum and minimum force	Soft pulp and skin
Merlot (B)	-	+	High maximum force	Firm skin
Pinot blanc (W)	++	+	Low maximum force	Soft skin
Pinot gris (W)	++	+	Low maximum force	Soft skin
Sauvignon blanc (W)	+++	++	Low maximum force, low deformation at maximum and minimum force	Soft pulp and skin
Syrah (B)	+	+	Mid-high maximum force, high deformation at maximum and minimum force	Variable skin firmness and consistent pulp
Trebbiano di Soave (W)	-	-	High maximum force, high deformation at maximum and minimum force	Firm and turgid berries

White-yellow (W) and blue-black cultivars (B) are indicated. Level of infestation: - <15%, + 15–30% ++ 30–50%, +++>50%; Level of emergence: - <1%, + 1–10%<, ++>10%. *In the present analyses, the term skin represents the epidermis and most external mesodermic tissue layers.

### Drying grapes

#### Grape susceptibility to D. suzukii infestation

As for ripening berries, also for drying grapes the percentage of infested berries was used as proxy for infestation level, because both number of infested berries and number of eggs laid per berry resulted highly correlated (n = 12, df=46, p-value<0.0001, r^2^ = 0.38).

**Figure 4:**
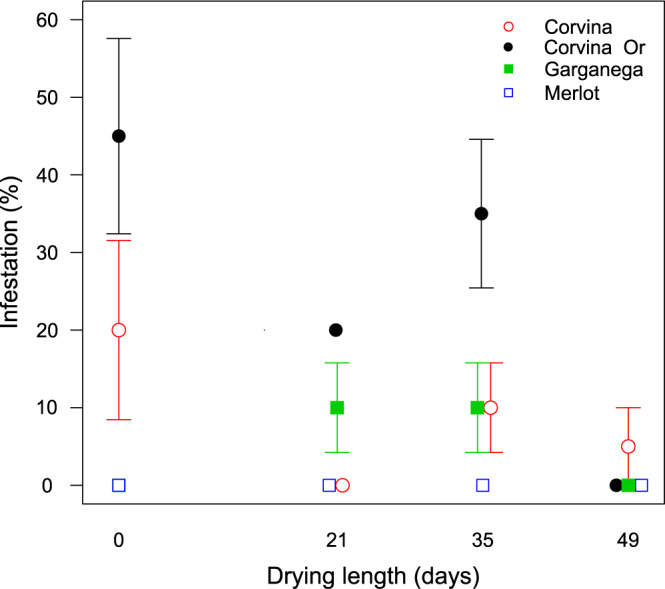
Infestation rate in the tested cultivars during the drying period. Corvina was also harvested overripe (Or). Error bars show standard error.

Merlot was never infested and discarded in the following analyses (Fig. 4). During the drying process, the grape cultivars presented differences in infestation over time (interaction time*cultivar F_4:27_ = 3.29, p = 0.025; Fig. 4). Garganega had low infestation levels at all the time points measured: less than 10% of berries infested on average, varying up to 20%. Corvina differed according to ripening level at harvest: overripe grapes (Or) were more susceptible to infestation than ripe ones, reaching values of 60% of infected berries (Fig. 4). At the end of the experiment, after 49 days of drying period, all cultivars resulted in low or zero infestation.

During the drying process, adults developed only from overripe grapes of Corvina cultivar with a high rate (Table 3).

**Table 3:** Total number of laid eggs, total number of emerged adults, and the resulting rate of emergence during the drying period.

Grape cultivar	Total number of eggs	Total number of emerged adults	Emergence rate (%)
Corvina Overripe (B)	34	4	11.8
Corvina (B)	4	0	0.0
Garganega (W)	23	0	0.0

Cultivars sorted by descending rate of emergence. White-yellow (W) and blue-black cultivars (B) are indicated. Merlot was discarded due to the zero-infestation recorded during the trials.

#### Texture and chemical features

In the drying process, texture features varied among cultivars over time. It is interesting to note that maximum force remained stable during the whole drying period, while deformation at maximum force progressively increased with the dehydration of berries. Instead, the chemical features remained more constant during the drying period with the exception of sugar content that sizably increased (supplementary dataset).

#### Relationships among entomological-mechanical-chemical features

**Figure 5:**
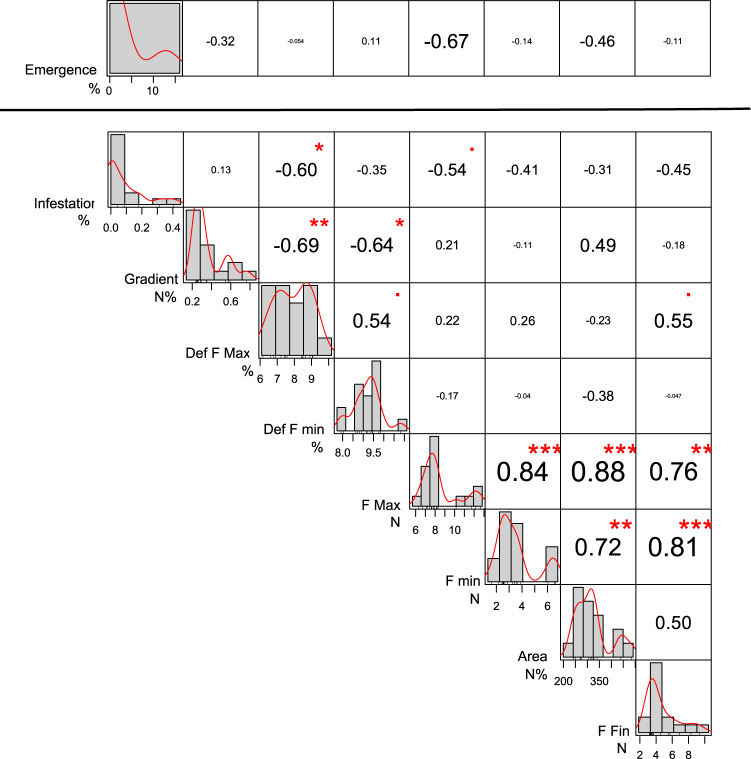
Correlation chart in the texture-entomological analysis for grapes during the drying process. The frequency distribution and kernel density estimation of each variable is shown on the diagonal. The value of the Pearson’s correlation (r) and significance level (p-value) corrected for multiple comparisons as asterisks (***<0.001, **<0.01, *<0.05, <0.10) are shown at the top of the diagonal; the size of the numbers is an indicator of the correlation value. The data range of values and units of measurement are given at the bottom of the diagonal. The analyses of emergence percentage (separated from the other data by a bold line) were performed only in samples where more than 1 egg was laid (n = 6).

Similarly to the ripening process, the drying period was characterized by a significant effect of berry texture features impacting the susceptibility to infestation (Fig. 5).

The infestation resulted correlated (P = 0.05) with deformation at maximum force and tended to inversely correlate with maximum force (P < 0.10; Fig. 5). The infestation percentage declined during the drying process as a result of the increase in deformation at maximum force. This pattern differed among the cultivars, ranging from the Corvina overripe grapes, characterized by low deformation and high susceptibility to infestation, to the Merlot which showed high deformation and no infestation (supplementary Fig. S3).

The emergence, analysed in a sub-sample where more than 1 egg was laid, showed no significant correlation with investigated features (Fig. 5).

Similarly to ripening grapes, none of the chemical parameters had any effect on infestation and larval development during drying period (supplementary Figure S4).

**Figure 6:**
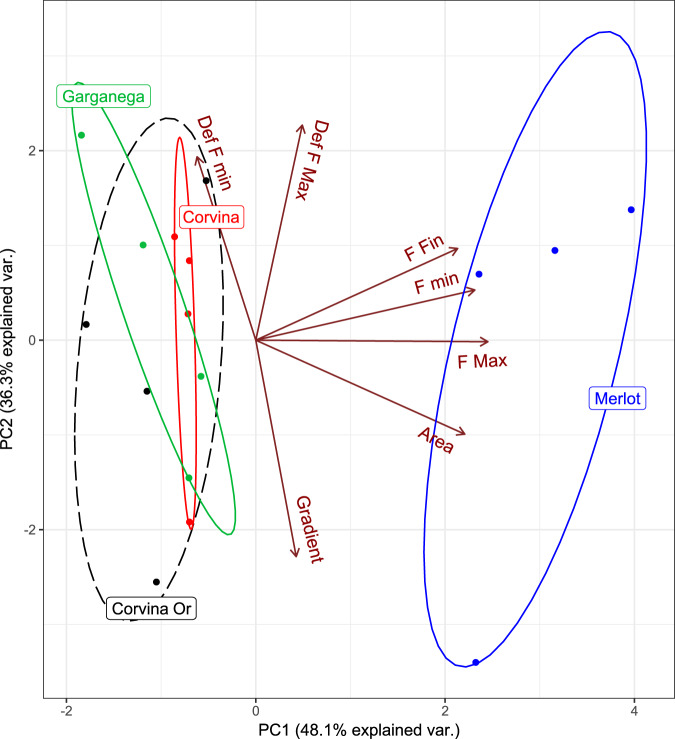
Spatial arrangement of mechanical texture features investigated in the PCA analysis for grapes during the drying process. Ellipses group the individual values of the cultivars (95% confidence).

The grouping of the four harvested samples is evident in the PCA 2D-plot (Fig. 6) performed to depict the investigated mechanical texture proprieties. The spatial distribution of the samples appears consistent with the variable orientation imposed on the PCA plot. Merlot is plotted towards higher values of area, minimum force, maximum force and final force. The other three cultivars cluster together opposite to the high forces and area values.

## Discussion

The suitability of *V. vinifera* for infestation by *D. suzukii* is well established, while the driver in the susceptibility of different cultivars remains uncertain^20^. Grapevine can be a host plant for this pest^12–15^ and damage has been found in European countries on different white and black grape cultivars^16,23,24^. On the other hand, studies conducted by Lee *et al*.^13^, Bellamy *et al*.^26^ and Cai *et al*.^38^ found low attraction for grapes, suggesting that *D. suzukii* may prefer fruit with a thin skin. In addition, laboratory studies on grape berries reported low oviposition, slow developmental rates and limited survival to the adult stage^13,17–19^.

However, the infestation of *D. suzukii* in a vineyard determines economic losses and represents a sizable threat for quality wine production^16,23,24,27^. This apparent discrepancy can be explained by the different susceptibility to *D. suzukii* infestation of the various grape cultivars. It becomes pivotal to estimate such susceptibility to forecast economic repercussions of infestation and plan pest control strategies. Many aspects may contribute to the susceptibility for infestation in orchards such as fruit attractiveness (e.g. colour, odour and short-wavelength reflectance), microclimatic conditions, landscape characteristics and cultural practices (e.g. cultivar selection, plant nutrition, soil tillage, insecticide applications) and *D. suzukii* characteristics such as temperature-induced plasticity^11,13,16,18,25,26,31,39–47^. These aspects can also influence the transferability of laboratory results to the field situations^22^.

However, the essential element for infestation is reasonably the set of intrinsic characteristics of the berries since these features shape the fundamental micro-environment for egg oviposition and larval development. Thus, to determine whether the different susceptibility to infestation and adult emergence across grapevine cultivars is influenced by chemical and mechanical texture features of the berries, we tested twelve cultivars of renowned economic value during the ripening period useful for all winemaking productions. In addition, for three cultivars, the study was extended to the subsequent drying phase in a drying-loft for raisin wine production. The rates of infestation and emergence were related to chemical and mechanical texture features of the berries to explain the role of skin and pulp characteristics in determining the nature of the susceptibility.

The findings showed that the susceptibility to *D. suzukii* infestation, across the entire harvesting period, varies to a great extent among cultivars. Barbera, Garganega, Merlot and Trebbiano di Soave were infested only in a low percentage, thus showing the lowest level of susceptibility. Cabernet Sauvignon, Corvina and Syrah presented low levels of infestation (15% to 30% of berries infested on average). Chardonnay, Pinot blanc, and Pinot gris had medium-high infestation levels, up to 80% of infested berries. Glera and Sauvignon blanc presented higher levels, in some cases reaching complete berry infestation. Our results are partially comparable with those previously published on Chardonnay where 15.9% of infestation and 5.1% of emergence rate were found^27^ and on Cabernet sauvignon in open field conditions, characterized by average pest incidence of ~0.5 eggs/berry^31^. Differently from Ioriatti *et al*.^27^. where time showed an influence on susceptibility, in our experiment no significant changes in infestation were observed over time probably because our sampling period was placed in the last and more stable ripening phase, excluding, in particular the green stage of the fruit that might largely affect the variability of the forces, especially when it is measured as an end point value.

Furthermore, many studies underlined the role of chemical features of the grape berries in determining the rate of infestation^27,29,30,48^. However, these studies were addressed over the entire timespan of berry development from green to full ripening stage; thus, encompassing the major grapevine physiological transformations. Instead, our study focused specifically on the ripening period and reveals that the infestation and rate of emergence are not related to the analysed chemical factors (*i.e*. pH, sugar content and acidity) since these parameters remain stable and within the optimal range for *D. suzukii* development^13,28^. Other biochemical compounds, such us polyphenols, may have a role on larval development^49^.

On the other hand, we revealed that in the ripening period, characterized by the highest *D. suzukii* pressure, the texture features are primarily responsible for grape susceptibility to infestation. Indeed, berries with a firm and elastic skin and consistent pulp resulted less susceptible to *D. suzukii* oviposition. In addition, larval development is influenced by fruit texture, mostly due to the physical characteristics of the pulp and the consequent intrinsic forces.

Grape texture has been investigated in the last decade because of its importance in several contexts. Variety textural profile is important in grape blanching processes^32^, since its changes are closely related to cell-wall structure. Texture can affect processing events, like drying and fermentation^50,51^, but it also strongly influences sensory quality and consumers’ satisfaction when the fresh product is consumed^52,53^.

The rate of infestation resulted primarily influenced by toughness of the external tissue layers, like epidermis and hypodermis through the exocarp (expressed by maximum force), skin elasticity (gradient) and pulp consistency (deformation at minimum force); together these texture features have a sizable influence on cultivar susceptibility.

The most susceptible cultivar to *D. suzukii* infestation are Glera and Sauvignon blanc, which are characterized by low values of maximal force and consequently by soft berries. Chardonnay, Pinot blanc, and Pinot gris showed a medium-high infestation level. In detail, berries of these cultivars have a soft skin and low skin elasticity, that likely ease egg laying by *D. suzukii*. In Cabernet sauvignon, characterized by low skin elasticity, *D. suzukii* females lay eggs easily despite the relative high maximum force (firmer berry). On the contrary, Merlot berries, which present a tough skin, showed low infestation rate, similarly in Barbera which is characterized by high skin elasticity. In Corvina, Garganega, Syrah and Trebbiano di Soave the low infestation was related to mid-high or high skin firmness together with high skin elasticity.

The subsequent larval development is negatively correlated to skin and outer tissue layers elasticity and positively by pulp consistency, indicating that texture features influence the rate of success for the emergence of new adults. In detail, from our analysis the high deformation, as proxy for consistent pulp, is negatively correlated to the emergence, while high maximal force and high gradient, indices of low elastic skin, are positively correlated to pest development. Specifically, the highly deformable and elastic Barbera and Trebbiano of Soave likely prevent the development of eggs into new adults. The high values of deformation at maximum and minimum force, mainly related to the internal pulp structure recorded in Corvina and Syrah can explain the low emergence rate. Cabernet sauvignon, Chardonnay, Garganega, Glera and Sauvignon blanc, characterized by low values of these deformations, may grant high eggs development. Elastic skins and pulps can have a negative effect on the respiratory process of eggs and young larvae, possibly limiting the gas exchanges of preimaginal stages.

The cultivar selection operated by *D. suzukii* in the field can be also linked to other factors such as skin colour, with a clear preference for the red cultivars, and berries health condition^18,22,27,44,46^. In addition, biochemical molecules as those of odours released by the grape colonizing microorganisms may stimulate *D. suzukii* egg deposition^20–22,44,54–58^. Further studies should address whether the mechanical texture features of berries could influence the berries healthiness (e.g. development of damage or micro cracks) and the growth of such microorganisms.

Infestation may also occur in the drying loft, increasing the vulnerability of grape bunches to pathogens. Our results demonstrate that not all the tested cultivars are susceptible to *D. suzukii* infestation. In fact, oviposition never occurred on Merlot possibly because this cultivar is characterized by tough skin and consistent pulp. For the other cultivars, infestations during the drying process were lower than during ripening, reaching a maximum 60% of infested berries. Again, texture features contribute to the susceptibility of drying grapes to *D. suzukii* infestation. In particular, the maximum force and deformation at maximum force, indexes of skin and pulp consistency, represent the most effective obstacle to oviposition. Thus, firm but highly deformable berries tend to be less susceptible than those that were more turgid. Unlike in the ripening process, the duration of drying becomes a relevant factor in determining infestation rate. Indeed, higher infestation occurs during the early drying phase when the berries are swollen. The dehydration of berries associated with progressive drying decreases the *D. suzukii* capacity to lay eggs.

The effect of the softening of berries during drying is evident also on the low emergence rate since infested berries produced adults only in Corvina overripe sample.

In conclusion, our study demonstrates that the texture features of berries play a role in the susceptibility of grapevine to *D. suzukii* infestation and influence the *D. suzukii* oviposition and emergence success during the ripening process.

In addition to oenological and terroir facets, viticultural zoning programmes should include cultivar susceptibility to infestation in order to meet the level of pest pressure with the proper grapevine cultivar. It thus becomes worthwhile, when planning new vineyards to prefer less susceptible cultivars in vineyards subjected to major pest pressure, which are generally those exposed to high spillover from semi-natural habitats^11,24,39,40^. In addition, when implementing Integrated Pest Management strategies, chemical treatments can be limited and targeted to the vulnerable cultivars only^41,59^.

Regarding the drying process, the increase of skin elasticity, which also includes the sub-epidermis tissue layers, that progressively hampers *D. suzukii* oviposition, suggests adapting the environmental drying loft conditions in order to induce a rapid dehydration of berries. Even anticipating the grape picking, as in the case of the Corvina samples, can help in reducing the further development of *D. suzukii* in the drying loft. However, both these suggestions must be adapted to the particular grape characteristics and the oenological goals.

## Methods

### Collection of ripening grapes

Twelve highly valuable grape cultivars were investigated (Table 4). The grapevines, eleven years old, were growing in the experimental vineyard of the Soave wine cellar “Borgo Rocca Sveva” (45°25′10.4″N, 11°15′06.1″E) trained with Guyot system at a plant distance 2.5 m between rows and 0.8 within rows. Agronomic and phytosanitary practices were according to the local IPM programme (Veneto Region - Phytosanitary service)^60^; no insecticides were applied during the trial period.

**Table 4:** Details of the twelve grape cultivars selected for the experiments.

Cultivar	Clone	Skin colour^1^	Rootstock
Barbera	FEDIT 3	deep blue (B)	SO4
Cabernet sauvignon	ISV F6	blue – black (B)	41B
Chardonnay	ENTAV 95	golden yellow (W)	SO4
**Corvina**	ISV CV 48	violet – blue (B)	41B
**Garganega**	ISV CV 84	amber yellow (W)	SO4
Glera	ISV ESAV 19	golden yellow (W)	SO4
**Merlot**	ENTAV 184	blue – black (B)	41B
Pinot blanc	LB 16	golden yellow (W)	SO4
Pinot gris	ENTAV 457	gray – violet (W)	SO4
Sauvignon blanc	CL 297	golden green (W)	SO4
Syrah	ENTAV 174	Blue (B)	SO4
Trebbiano di Soave	VITIVER 37	golden green (W)	SO4

^1^ skin colour from Calò *et al*.^70^; white-yellow (W) and blue-black cultivars (B) are indicated. Cultivars tested also for raisin wines are in bold.

Berries used in the bioassay were randomly collected from different vines and grapes. For each cultivar, samplings in the vineyard were performed weekly, from September 01^st^ to October 13^th^, 2015 covering the typical harvesting periods (supplementary Table S1) and those for all winemaking productions (e.g. wine from late harvest, wines rich in tannins and spicy overtones, with complex, baked fruit or honeyed aroma) as suggested by the local vineries.

### Collection of drying grapes

Boxes with grape bunches of three different cultivars (Corvina, Garganega and Merlot) were placed in a drying loft (located in Cazzano di Tramigna; 45°27′43.8″N, 11°12′20.6″E) to monitor infestation susceptibility and intrinsic characteristics during the drying process. Corvina, Garganega and Merlot grapes were harvested on September 28^th^. An additional overripe sample of Corvina was harvested on October 5^th^ in order to encompass the variability of ripening stages that characterize the oenological uses of this cultivar. Samples of drying grapes were collected three times during the drying process: 21, 35, and 49 days after harvest.

#### Drosophila suzukii infestation

Experimental infestations of grape berries were artificially conducted in the laboratory at DAFNAE Department of the University of Padua, located in Legnaro (Italy). Experimental units were composed of five healthy and fully intact berries, randomly selected among more than 100, placed inside 7 × 7 × 7 cm net cages. The berries were sustained by their racemes to ensure the durability and integrity of fruit conditions. Each berry was previously checked under a stereo-microscope to exclude the presence of previous eggs, larvae and/or physical damage. Five females and two males of *D. suzukii*, 6 to 8 days-old were released inside the cages. The *D. suzukii* cohort used for the experiments was drawn from a long-lived colony raised in the DAFNAE laboratory and originated from adults collected from cherry, blueberry and grape in Verona district (Italy), as described in Tonina *et al*.^61^. Flies were nourished with a 10% sugar aqueous solution supplied in 2 ml plastic tubes stoppered with cotton. This unit was replicated four times for each cultivar. All flies were removed after 24 h of contact with berries and the presence of dead individuals was checked. The number of eggs laid on each berry was counted under stereo-microscope. Only the berries containing eggs were subsequently stored in plastic containers (100 ml) and kept in a climatic chamber at 22 °C, ~70% relative humidity, and a 16:8 hours (L:D) photoperiod. These berries were checked twice a week to assess adult emergence; the numbers of newly emerged adults were counted during the three following weeks.

### Chemical analysis on grapes

Berries, from both the vineyard and the drying loft, were stored for no longer than 24 hours after collection at room temperature (21 ± 2 °C) before analysing. Physiological parameters were concurrently determined at each sampling on healthy berries for each tested cultivar by measuring the sugar content (°Brix) with a digital DBR35 refractometer, total acidity (meq/100 g) and pH using a titration Compact Titrator (Crison, Modena, Italy).

### Texture measurements

A penetrometer texture analysis was chosen in order to mimic the oviposition activity of the pest and inferring the tissue and cellular opposing resistance to the insect ovipositor penetration and egg development in the fruit. For this reason, compression analyses were not considered because they mainly rely on the deduced indexes that we thought were not representative for our purpose. The texture analysis was performed with a Zwick texture analyser, Zwicki model (Zwick Roell Italia, Genova). All experiments were conducted at room temperature (21 ± 2 °C). For each replicate, twenty berries were analysed resulting in 20 individual measurements. Texture penetrometer tests were carried out as in Giongo *et al*.^62^, with a 12.56 mm^2^ area cylindrical probe, penetrating up to 99% fruit deformation at 0.3 mm/s and an accuracy of 0.001 N. Force vs. deformation curves were recorded and flesh rupture mean force penetration was calculated and corresponded to firmness expressed in Newton. Stress vs. strain curves were analysed and the gradient was obtained from the slope of the loading curve at the point of its highest gradient (supplementary Figure S5). Seven texture describing mechanical features were used to infer variability and genotypic profiles, as described in Table 5. All measurements were processed through the TextXpert II software, coupled to the machine.

**Table 5:** List of the main mechanical features related to texture profiling, relative characteristics and characteristic of berry influencing each feature.

Mechanical features	General description	Unit	Acronym	Berry texture characteristics
Maximum force	Maximum force value recorded over the probe’s travel	N	F Max	Skin and external tissues firmness/fracturability
Maximum force deformation/strain	Computation of the maximum force associated with the curve on its whole length	%	Def F Max	Berry pulp consistency/firmness
Minimum force	Minimum force value recorded over the probe’s travel	N	F min	Berry pulp consistency/firmness
Minimum force deformation/strain	Computation of the minimum force associated with the curve on its whole length	%	Def F min	Berry pulp consistency/firmness
Final force	Force measured at the end of the probe’s travel	N	F Fin	Whole berry consistency/firmness
Area	Area underlying the mechanical profile	Nmm	Area	Whole berry consistency/firmness
Gradient	Young’s modulus or elasticity modulus, computed as ratio between stress and strain	MPa	Gradient	Skin and outer tissue layers elasticity

### Statistical analysis

All analyses and visualisations were implemented in R 3.4.1^63^. To test the correlation between the percentage of infested berries and the number of eggs laid per berry, a Pearson’s correlation was used applying the function “cor.test” from the package “stats”^64^.

Through individual linear models the effects of grapevine cultivar over time on the rate of infestation, in both ripening and drying grapes, were tested. In each model, cultivar and data of collection (or drying length) were entered as categorical and linear factors respectively. For these analyses, the raw entomological datasets were used (see supplementary dataset). Percentage of infested berries was arcsine square-root transformed prior to analyses. In preliminary analyses, chemical features such as sugar content, pH and total acidity were tested to line up and monitor the progress of maturation. The assumptions of the models were evaluated by inspecting diagnostic plots of model residuals. The analyses were performed using the functions “lm” and “anova” from the package “nlme”^65^.

Correlation charts were conducted separately for ripening and drying grapes using the full entomological and chemical or texture mechanical data set (see supplementary dataset). Correlation chart reports the frequency distribution and kernel density estimation of each variable, the value of the Pearson’s correlation (r) and significance level (p-value) adjusted for multiple comparisons using Benjamini–Hochberg (BH) equation^66^. Correlation charts and relative analysis were performed using functions “chart.Correlation” and “corr.test” from the packages “PerformanceAnalytics”^67^ and “psych”^68^ respectively.

Principal Component Analysis (PCA) were conducted separately for ripening and drying grapes with the full texture mechanical data set (see supplementary dataset), using functions “prcomp” and “ggbiplot” from the packages “stats”^64^ and “ggbiplot”^69^ respectively.

Preliminary analyses were performed to determine variables with low loading on components.

## Supplementary information

Supplementary information.

Supplementary information 2.

## Data Availability

All data generated or analysed during this study are included in this published article (and its Supplementary Information files).
