# Supplementary material for: Texture features explain the susceptibility of grapevine cultivars to Drosophila suzukii (Diptera: Drosophilidae) infestation in ripening and drying grapes
Source: Sci Rep. 2020 Jun 24;10:10245. doi: 10.1038/s41598-020-66567-9 (PMC7314830; doi:10.1038/s41598-020-66567-9)
Supplement: Supplementary file 2 — Supplementary information 2. [file 41598_2020_66567_MOESM2_ESM.pdf]

Texture features explain the susceptibility of grapevine cultivars to *Drosophila suzukii* (Diptera: Drosophilidae) infestation in ripening and drying grapes.

Lorenzo Tonina[1*], Folco Giomi[1], Manuel Sancassani[1], Matteo Ajelli[2], Nicola Mori[1,3], Lara Giongo[2]

[1]*Università di Padova - Legnaro (Pd), Italy - Department of Agronomy, Food, Natural resources, Animals and Environment (DAFNAE);*

[2]*Fondazione Edmund Mach - San Michele all'Adige (Tn), Italy - Genomics and Biology Fruit Crops Department, Research and Innovation Centre*

[3]*Università di Verona - Italy - Department of Biotechnology;*
* Corresponding author

E-mail: lorenzo.tonina@gmail.com, Tel.: +39 340 2876514, Fax: +39 049 8272784

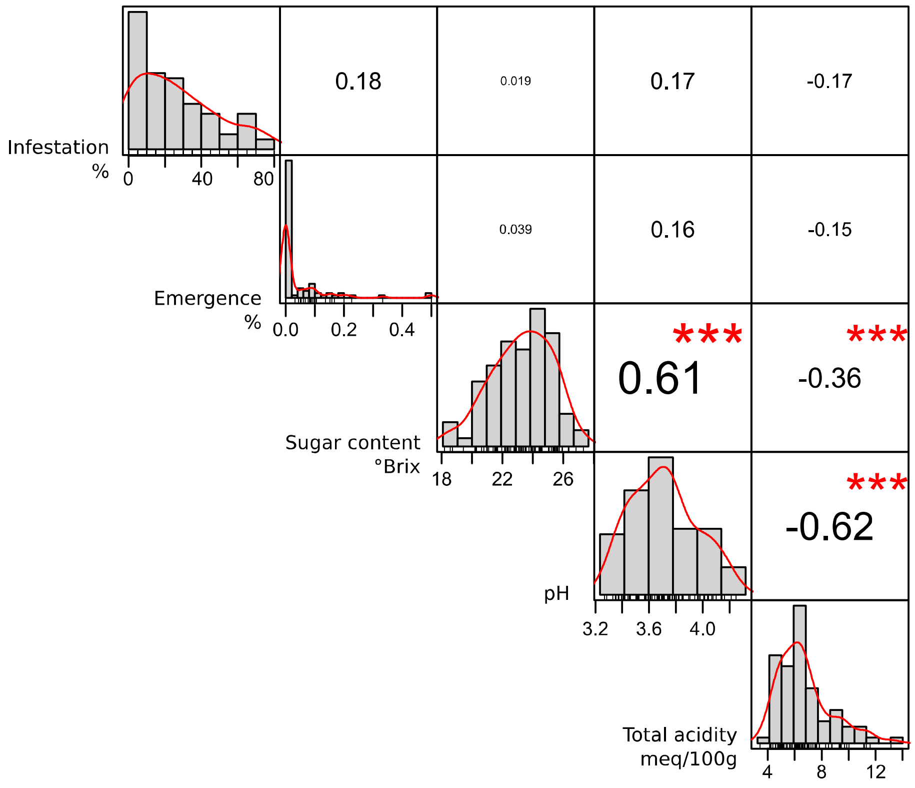

Supplementary Figure S1. Correlation chart in the chemical-entomological analysis for ripening grapes. The frequency distribution and kernel density estimation of each variable is shown on the diagonal. The value of the Pearson's correlation (r) and significance level (p-value) adjusted for multiple comparisons as asterisks (***<0.001, **<0.01, *<0.05, .<0.10) are shown at the top of the diagonal; the size of the numbers is an indicator of the correlation value. The data range of values and units of measurement are given at the bottom of the diagonal.

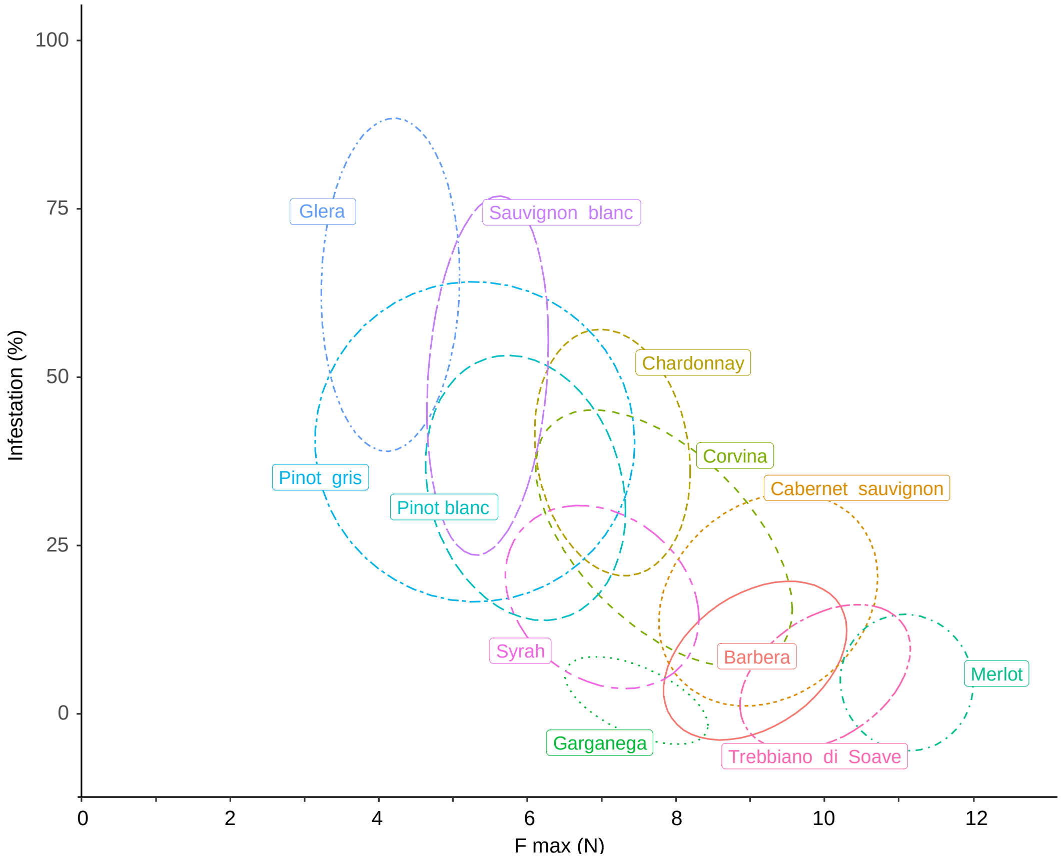

Supplementary Figure S2. Relationship between infestation level and maximum force applied to the penetration. Ellipses group the individual values of cultivars (67% confidence). This plot shows the influence of exocarp resistance in determining the infestation level of each cultivar.

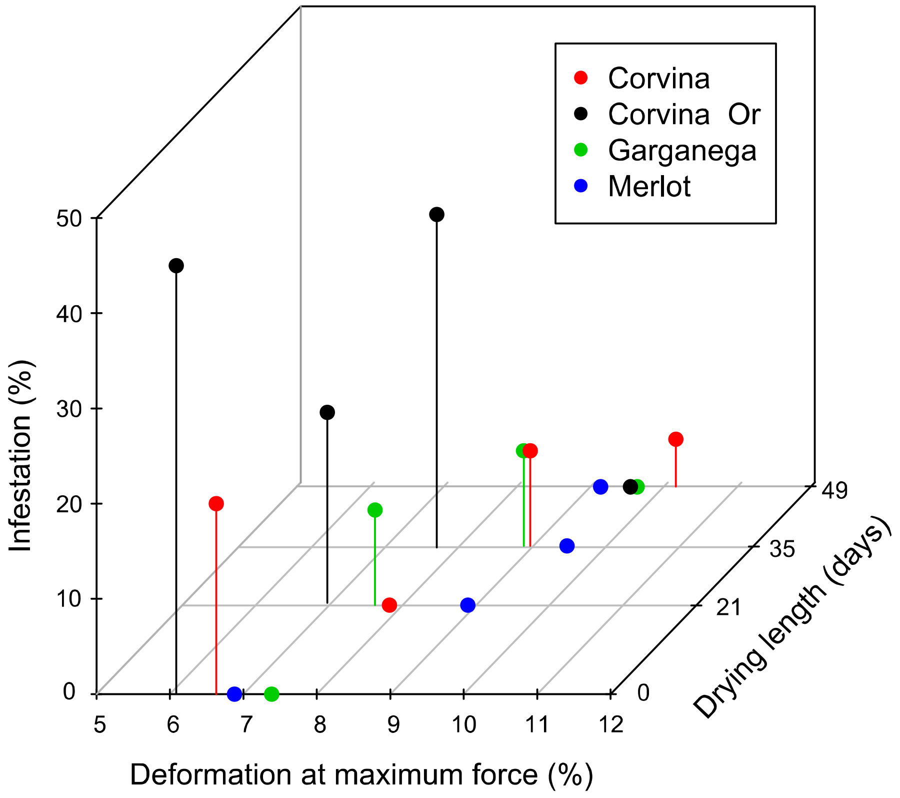

Supplementary Figure S3. *Drosophila suzukii* infestation in relation to deformation at maximum force, drying length in the three cultivars investigated in the drying period. Corvina was also harvested overripe (Or).

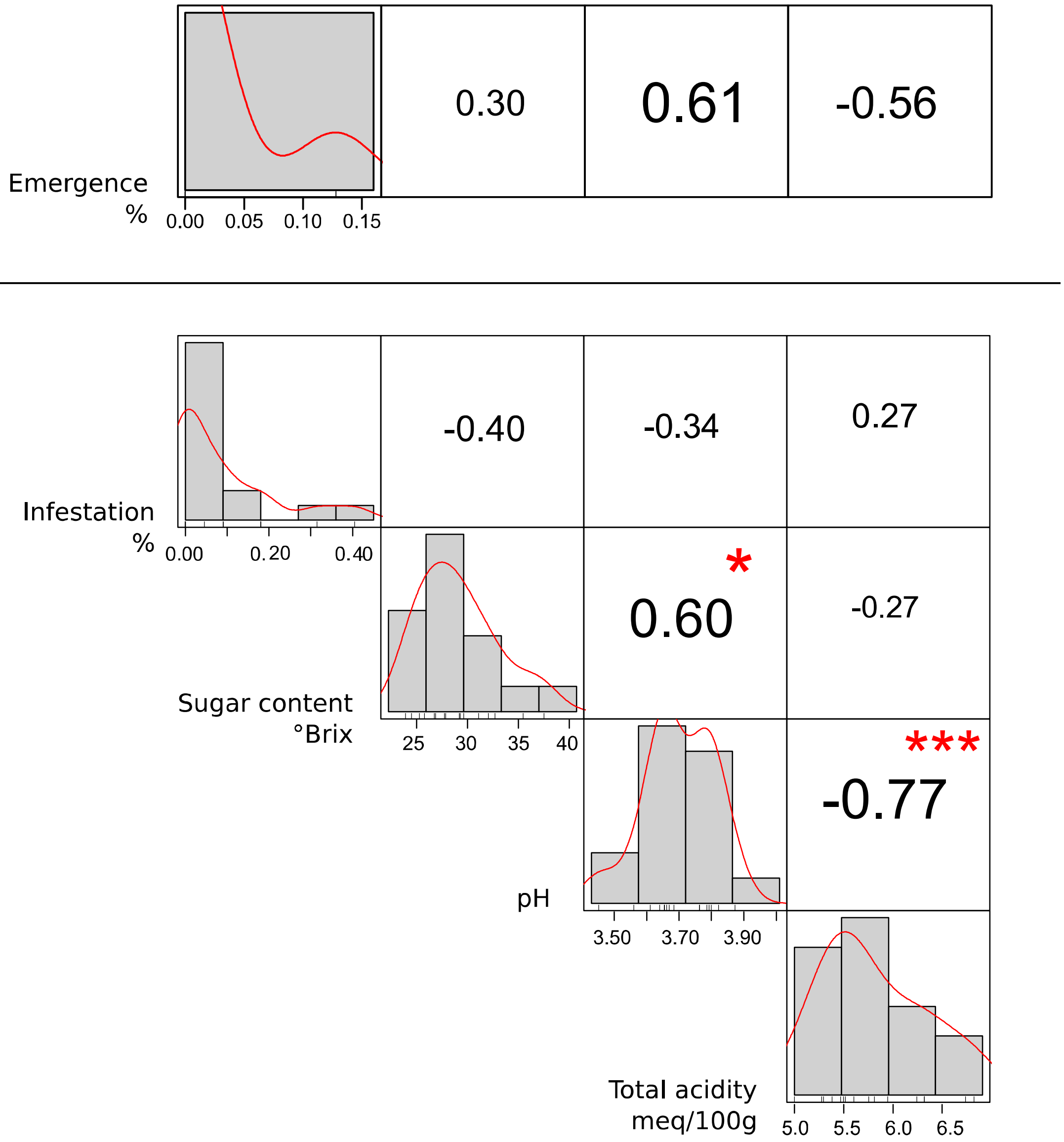

Supplementary Figure S4. Correlation chart in the chemical-entomological analysis for grapes during the drying process. The frequency distribution and kernel density estimation of each variable is shown on the diagonal. The value of the Pearson's correlation (r) and significance level (p-value) adjusted for multiple comparisons as asterisks (***<0.001, **<0.01, *<0.05, .<0.10) are shown at the top of the diagonal; the size of the numbers is an indicator of the correlation value. The data range of values and units of measurement are given at the bottom of the diagonal. The analyses of the percentage of emergence (separated from the other data by the bold line) were performed only in samples where more than 1 egg was laid (n=6).

Cultivar	Ripening categories	Harvesting period
Barbera	medium	September 10-30
Cabernet sauvignon	medium late	September 20-30
Chardonnay	early - medium early	August 20 - September 10
Corvina	medium late	September 20-30
Garganega	medium	September 10-30
Merlot	medium late	September 20-30
Pinot bianco	early - medium early	August 20 - September 10
Pinot grigio	medium early	August 20 - September 10
Glera	medium late	September 10-30
Sauvignon bianco	medium early	September 1-10
Syrah	medium	September 10-30
Trebbiano di Soave	medium	September 10-30

Supplementary Table S1. Ripening categories from Calò et al.,[70] and typical harvesting period in Veneto in 2015 for grape cultivars. Harvest can be early or late according to the oenological purposes (e.g. wine from late harvest, wines rich in tannins and spicy overtones, with complex, baked fruit or honeyed aroma).

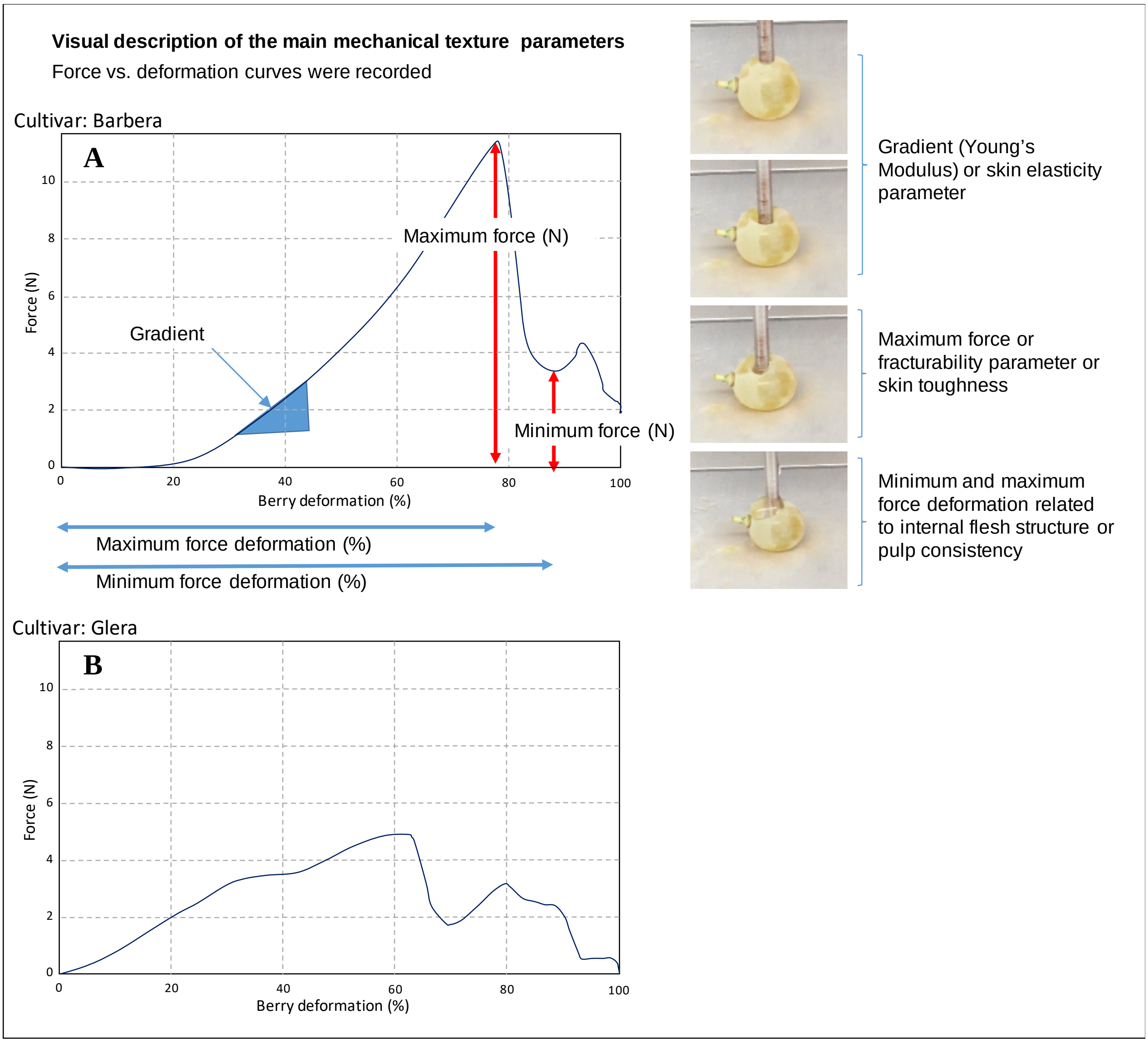

Supplementary Figure S5. Stress vs. strain curve in Barbera (A) and Glera (B) cultivars. Gradient, forces and deformations are highlighted in the curve A and in berry penetration.
